# Delayed recovery of non-marine tetrapods after the end-Permian mass extinction tracks global carbon cycle

**DOI:** 10.1098/rspb.2011.1895

**Published:** 2011-10-26

**Authors:** Randall B. Irmis, Jessica H. Whiteside

**Affiliations:** 1 Utah Museum of Natural History, 1390 E. Presidents Circle, Salt Lake City, UT, 84112-0050, USA; 2 Department of Geology and Geophysics, University of Utah, Salt Lake City, UT 84112-0102, USA; 3 Department of Geological Sciences, Brown University, 324 Brook Street, Box 1846, Providence, RI 02912, USA

**Keywords:** richness, evenness, relative abundance, Synapsida, Archosauromorpha, chaotic carbon interval

## Abstract

During the end-Permian mass extinction, marine ecosystems suffered a major drop in diversity, which was maintained throughout the Early Triassic until delayed recovery during the Middle Triassic. This depressed diversity in the Early Triassic correlates with multiple major perturbations to the global carbon cycle, interpreted as either intrinsic ecosystem or external palaeoenvironmental effects. In contrast, the terrestrial record of extinction and recovery is less clear; the effects and magnitude of the end-Permian extinction on non-marine vertebrates are particularly controversial. We use specimen-level data from southern Africa and Russia to investigate the palaeodiversity dynamics of non-marine tetrapods across the Permo-Triassic boundary by analysing sample-standardized generic richness, evenness and relative abundance. In addition, we investigate the potential effects of sampling, geological and taxonomic biases on these data. Our analyses demonstrate that non-marine tetrapods were severely affected by the end-Permian mass extinction, and that these assemblages did not begin to recover until the Middle Triassic. These data are congruent with those from land plants and marine invertebrates. Furthermore, they are consistent with the idea that unstable low-diversity post-extinction ecosystems were subject to boom–bust cycles, reflected in multiple Early Triassic perturbations of the carbon cycle.

## 1. Introduction

The end-Permian mass extinction event at approximately 252.6 Ma [1] is the largest mass extinction in Phanaerozoic Earth history in terms of diversity loss [2,3]. This event caused a permanent restructuring of marine and terrestrial ecosystems [4,5] that set the stage for the origin of modern biotas. Detailed examination of the marine fossil record demonstrates that these ecosystems took 5–8 million years to recover; not until the early Middle Triassic (Anisian) are diversity and complexity comparable with that of pre-extinction faunas [6–8]. The cause of this mass extinction has not been fully resolved, but multiple lines of evidence point to greenhouse gases and other compounds from the Siberian Traps [1,6,9,10] as a trigger. These data suggest major environmental stress during and immediately after the extinction event [11–13].

Connected with this environmental stress, the global carbon cycle displays major perturbations associated with the end-Permian extinction, beginning with a large-scale initial negative excursion, followed by multiple positive–negative couplets throughout the Early Triassic [7,8,14]. This ‘chaotic carbon interval’ [8] did not stabilize until the early Anisian (approx. 246–245 Ma), at the same time that marine ecosystems also recovered. Many authors have interpreted this chaotic carbon interval to represent multiple inputs of volcanogenic greenhouse gases [15] or an otherwise unstable palaeoenvironment [7,14]. However, one of us (J.H.W.) has recently suggested that these chaotic carbon intervals reflect ecosystem instability itself, caused by boom–bust cycles with repeated collapse owing to low redundancy in trophic and functional networks [8]. This interpretation is also consistent with both the low diversity throughout the Early Jurassic in the aftermath of the end-Triassic extinction [6,8] and Permo-Triassic ecological models [16,17].

Although well investigated in the marine fossil record, one of the major outstanding questions about the end-Permian mass extinction is how it affected terrestrial ecosystems. There is little doubt that a synchronous extinction event occurred on land [18], but how severe was it? The few detailed studies of Permo-Triassic terrestrial floras agree that they were affected by the extinction [19–23], but there is major disagreement over the severity of the extinction [24]. Independent of floral evidence, there is strong evidence for environmental stress on land [25–27].

Data are equally limited for non-marine vertebrates across the Permo-Triassic boundary. A number of studies have investigated the first and last appearances of individual lineages across the boundary [28–32], broadly agreeing that vertebrates were affected by the extinction, but reaching different conclusions on how quickly vertebrate faunas recovered. Few studies have investigated non-marine vertebrate taxonomic richness, relative abundance, and evenness across the extinction and recovery interval. Those that have been published have reached conflicting conclusions; Pitrat's [33] richness data indicated only a minor event at the end-Permian, whereas King [34,35] saw a larger effect on vertebrate assemblages, but concluded it was part of a longer gradual decline during the Late Permian. Fröbisch [36] examined species richness of the synapsid clade Anomodontia (dicynodonts and relatives), and determined that they were severely affected by the end-Permian extinction, even when accounting for geological sampling bias. Using a non-taxonomic approach, the analysis of Sahney & Benton [5] concluded that non-marine ecological guilds were severely affected by the end-Permian extinction event and did not recover until the Late Triassic.

The goal of our study is to elucidate the effect of the end-Permian extinction on terrestrial ecosystems, focusing on non-marine vertebrate faunas. By using specimen-level data, we ask what is the pattern of vertebrate richness, relative abundance and evenness across the Permo-Triassic boundary? Are these data consistent with an unstable ecosystem recorded by the Early Triassic chaotic carbon interval [8], a hypothesis supported by modelling of Early Triassic terrestrial vertebrate ecosystems [17]? This is the first study to examine evenness for non-marine vertebrates across the Permo-Triassic boundary, and to look at all metrics with an explicit eye towards sample standardization and quantitative evaluation of potential biases.

## 2. Methods

### (a) Choice of datasets

To investigate non-marine vertebrate palaeoecology across the Permo-Triassic boundary, we chose two published regional specimen-level datasets identified to genus. These data are ideal because they allow for easy sample-size standardization (i.e. by number of specimens), lessen problems with long-distance stratigraphic correlation and allow identification of any regional differences in patterns (in contrast with global analyses). Our main dataset comes from the Karoo Basin of southern Africa [37] and includes hundreds to thousands of specimens per temporal bin (see the electronic supplementary material); we removed erroneous occurrences mentioned by the original authors and did not include generically indeterminate records except in clade-level relative abundance analyses. We also used a second dataset from the Ural region of Russia [29], though the number of analyses we could conduct with these data was limited because of lower sample sizes (approx. 40–100 specimens per temporal bin; see the electronic supplementary material).

The temporal bins for our analyses were biostratigraphic zones identified by the original authors [29,37]. The correlation of these zones to the Permian and Triassic timescale follows Rubidge [38] for southern Africa and Benton *et al*. [29] for Russia. The absolute ages and durations for the Global Stratotype Section and Point-defined stages of the timescale follow the revisions of Walker & Geissman [39] and Mundil *et al*. [40].

### (b) Analyses

**Figure 1.**
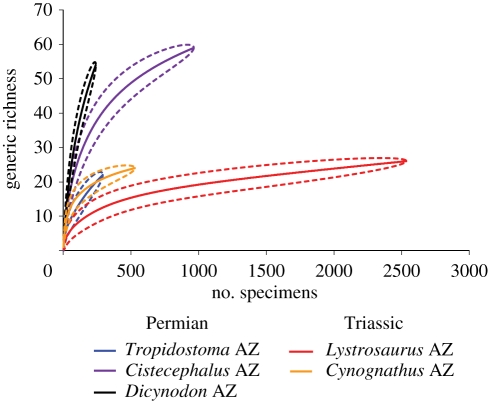
Rarefaction curves of generic richness versus number of specimens for five Permo-Triassic assemblage zones (AZs) in the Karoo Basin of southern Africa. Dashed lines represent 95% confidence intervals.

**Figure 2.**
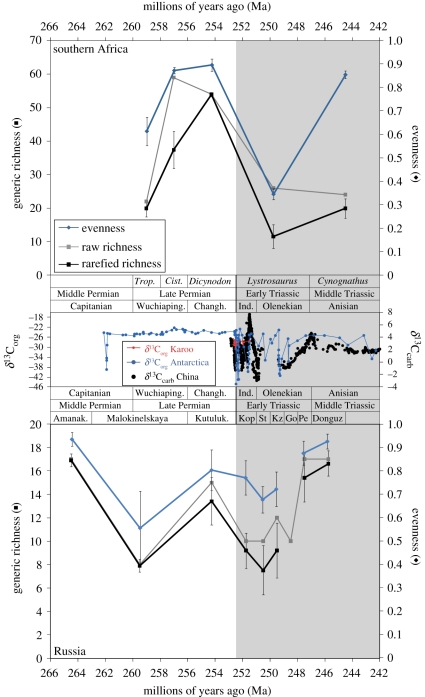
Comparison of raw generic richness, rarefied generic richness and evenness for non-marine tetrapods from the Permo-Triassic of southern Africa and Russia to carbon isotopic records as a proxy for the global carbon cycle. Vertically adjacent to each graph are the biostratigraphic divisions used for temporal bins in this analysis (AZs for southern Africa and svitas for Russia). See text for details on calculation of richness and evenness, and correlation with geological timescale. *δ*^13^C_org_ data from non-marine sections at Lootsberg Pass in the Karoo Basin of southern Africa [42] and Graphite Peak in Antarctica [43]. *δ*^13^C_carb_ data from marine sections in South China [6]. Amanak., Amanakskaya; Changh., Changhsingian; *Cist*., *Cisticephalus* Assemblage Zone; Go, Gostevskaya; Ind., Induan; Kop, Kopanskaya; Kz, Kzylsaiskaya; Kutuluk., Kutulukskaya; Pe, Petropavlovskaya; St, Staritskaya; *Trop*., *Tropidostoma* Assemblage Zone; Wuchiaping., Wuchiapingian.

**Figure 3.**
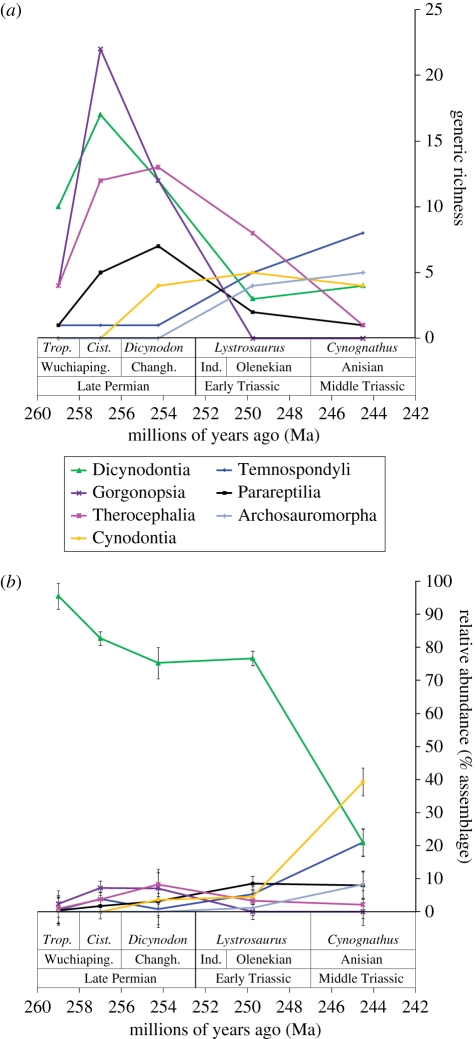
(*a*) Generic richness and (*b*) relative abundance for major tetrapod clades in the Permo-Triassic Karoo Basin of southern Africa. See text for details on calculation of richness and relative abundance, and correlation with geological timescale. Changh., Changhsingian; *Cist*., *Cisticephalus* Assemblage Zone; Ind., Induan; *Trop*., *Tropidostoma* Assemblage Zone; Wuchiaping., Wuchiapingian.

We investigated raw generic richness for each temporal bin (i.e. total number of genera) irrespective of sample size. These data were also rarefied using Analytic Rarefaction v. 1.3 [41] to standardize for sample size (figure 1). The southern African dataset was rarefied to 241 specimens and the Russia dataset to 48 specimens (figure 2); these levels were chosen based on the smallest sample size among the temporal bins. We also investigated taxonomic richness for each major clade of vertebrates (figure 3*a*; see the electronic supplementary material), but could not rarefy these data because of low sample sizes, and the fact that unequal relative abundance between clades might bias results. Evenness of assemblages (figure 2) was calculated using Olszewski's modification of Simpson's Index [44]; to account for sample-size differences, we calculated confidence intervals for this metric using the method of Davis [45], based on Simpson's original calculation of variance (see the electronic supplementary material). Finally, we explored the relative abundance of each major clade for each temporal bin (figure 3*b*) and accounted for sample-size differences by calculating confidence intervals using the method of Moore *et al*. [46] (see the electronic supplementary material).

To investigate possible taxonomic biases in our data, we revised the *Lystrosaurus* Assemblage Zone (AZ) data using recent publications (see the electronic supplementary material) and compared it with the original *Dicynodon* AZ data (see the electronic supplementary material). This constitutes a conservative test, because recent taxonomic revisions and newly described taxa should increase the generic richness of both time bins [47,48]. Therefore, revising only the post-extinction bin (*Lystrosaurus* AZ) should bias our data against finding a major extinction signal. If, despite this bias, we find strong evidence for extinction, it will show that our data are robust to taxonomic biases. These revised *Lystrosaurus* AZ data were only used for this taxonomic bias test (see the electronic supplementary material), and are not represented in figures 1–3.

Geological and sampling biases of palaeoecological data are a pervasive concern. Sample-size differences across temporal bins were accounted for through rarefaction, but we also evaluated other potential biases within our data. The amount of outcrop area that fossils are collected from can influence observed diversity; the greater the expanse of outcrop, the more likely one is to find additional specimens and taxa. We fitted a linear regression to the total outcrop and productive outcrop area estimates of King [35] versus total number of specimens, raw generic diversity and rarefied generic richness (see the electronic supplementary material). Similar outcrop data for the Russian dataset are unavailable. We also investigated whether the temporal duration of each bin affected diversity, as a longer temporal bin is likely to circumscribe a greater number of specimens and higher diversity of taxa. We fitted a linear regression to bin duration in millions of years versus raw generic richness for each temporal bin in both the southern African and Russian datasets (see the electronic supplementary material).

To investigate how non-marine vertebrate palaeoecology across the Permo-Triassic interval relates to the global carbon cycle, we make comparisons with two main isotopic datasets: a *δ*^13^C_carb_ record from shallow marine carbonates in South China, which spans the latest Permian through Middle Triassic [6]; and a *δ*^13^C_org_ record from non-marine siliciclastic strata at Graphite Peak, Antarctica, which spans the latest Permian through early Middle Triassic (Anisian) [43] (figure 2). Unfortunately, there are no long isotopic records from southern Africa or Russia, but we do make comparisons with a short *δ*^13^C_org_ record from the Karoo Basin that crosses the Permo-Triassic boundary [42] (figure 2).

## 3. Results

Despite sample sizes that range from less than 250 specimens to greater than 2500 specimens, the rarefaction curves show a clear difference in generic richness among southern African Permo-Triassic AZs (figure 1). The two Triassic bins (the *Lystrosaurus* and *Cynognathus* AZs) have much lower generic richness than the Late Permian *Cistecephalus* and *Dicynodon* AZs, even when accounting for sample size (figure 1). Somewhat surprisingly, the early Late Permian *Tropidostoma* AZ also has low generic diversity; although this bin historically has had lower collecting effort, our rarefied diversity estimate minimizes sample-size effects. Placing these data into stratigraphic sequence, both the raw and rarefied richnesses show a precipitous drop across the Permo-Triassic boundary (figure 2). Richness remains low during the Early Triassic; in southern Africa, richness is still depressed during the Middle Triassic *Cynognathus* AZ. By contrast, in Russia, richness appears to recover during the early Anisian (figure 2). In both the southern Africa and Russia datasets, generic richness during the early Late Permian is significantly lower than other Permian intervals, displaying values comparable to the Early Triassic temporal bins.

Evenness of these vertebrate assemblages shows similar patterns. Both in southern Africa and Russia, evenness declines across the Permo-Triassic boundary, but recovers by the early Middle Triassic (figure 2). In Russia, the decline across the Permo-Triassic boundary is not statistically significant, probably because of low sample sizes. Similar to the pattern in generic richness, evenness is also low in both southern Africa and Russia during the early Late Permian (figure 2). It also appears that in both regions, evenness recovers before richness during the Triassic Period. Only dicynodonts from southern Africa have a large enough sample size to investigate their clade-specific evenness patterns through time. These data demonstrate that southern African dicynodont evenness drops to near zero across the Permo-Triassic boundary because of the prevalence of *Lystrosaurus*, and does not recover during the Middle Triassic *Cynognathus* AZ (electronic supplementary material, figure S1).

Clade-specific generic richness for southern Africa also displays clear effects from the end-Permian mass extinction (figure 3*a*), with the caveat that these values cannot be sample-standardized because of low total sample sizes. These data display clearly the extinction of gorgonopsian synapsids across the Permo-Triassic boundary. Dicynodonts show a major decline in richness across the boundary, whereas the decline in parareptiles and therocephalian synapsids is more gradual and extends into the Middle Triassic (*Cynognathus* AZ; figure 3*a*). Both temnospondyl amphibians and archosauromorphs display a distinct increase in generic richness across the Permo-Triassic boundary, and this increase continues into the Middle Triassic (figure 3*a*).

The trends in relative abundance of individual clades for southern Africa are very different from those of richness. There are slight to moderate increases in the abundance of temnospondyls and parareptiles across the Permo-Triassic boundary, and similarly modest decreases in the relative abundance of gorgonopsians (which go extinct) and therocephalians, but all of these are either not statistically significant or only weakly so (figure 3*b*). Dicynodont abundance (relative to other tetrapod clades) does not change, probably because the latest Permian *Dicynodon* AZ and earliest Triassic *Lystrosaurus* AZ are dominated by their eponymous dicynodont genera; however, *Dicynodon* AZ dicynodont evenness may actually be significantly higher based on an ongoing taxonomic revision of *Dicynodon* [47]. The major changes in relative abundance occur between the Early Triassic *Lystrosaurus* AZ and Middle Triassic *Cynognathus* AZ. During this interval, dicynodont relative abundance decreases dramatically, from approximately 75 to approximately 20 per cent of specimens (figure 3*b*). In contrast, temnospondyl and cynodont abundance more than doubles; these two clades comprise more than 60 per cent of all specimens in the Middle Triassic *Cynognathus* AZ (figure 3*b*).

## 4. Discussion

### (a) Potential biases of observed patterns

Palaeoecological metrics like those reported above can correlate with a number of geological biases and other non-biotic controls. Thus, it is extremely important to investigate possible non-biological signals in any dataset; these primarily consist of sampling and geological biases, along with errors and other limitations among the original specimen data.

At the most basic level, sample size is a major control of diversity; greater sample size increases the potential to sample more taxa and clades. Conversely, low sample size can amplify stochastic effects, whereby a random draw is not representative of the total fossil assemblage; emerging methodological alternatives may help ameliorate this problem in the near future. For total generic richness, rarefaction minimizes unequal sample sizes across different temporal bins [49]. The confidence intervals we calculated for evenness [45] and relative abundance [46] metrics allow for conservative interpretation of these values across differing sample sizes. Low sample size for clade-specific richness meant that we could not rarefy these data, so they will be most sensitive to the biases described below.

Geological outcrop area is one major bias of palaeoecological datasets, because more widespread outcrop affords the opportunity to discover more specimens and taxa. Comparison of the available outcrop area for each southern African temporal bin with raw and rarefied richness for each bin indicates that there is a very weak to no correlation, and a moderate correlation with number of specimens (electronic supplementary material, figure S2*a*–*c*). In contrast, when the *Dicynodon* AZ is removed from the comparison between number of specimens and outcrop area, the relationship becomes highly significant (electronic supplementary material, figure S2*d*). This is because the *Dicynodon* AZ is particularly fossiliferous for its available outcrop area, with over 2500 specimens in our dataset (figure 1). Like outcrop area, a longer temporal bin duration can increase the number of specimens and observed richness. We observe a very weak inverse correlation (*R*^2^ = 0.1962) between bin duration and richness in southern Africa, but this is contrary to our prediction that longer time bins should have higher richness, and there is no significant correlation in the Russian dataset (electronic supplementary material, figure S3). Both of these biases are ameliorated by our rarefaction of the data, which has a weakly inverse correlation, contrary to our prediction that they would be directly correlated (see the electronic supplementary material).

Taxonomic opinion and error can also affect analyses of any palaeoecological dataset. There is always the chance that some specimens are misidentified, though diversity metrics are robust in the face of high taxonomic error rates, if this error is randomly distributed across the dataset [50]. Although this should be minimized as both databases were vetted by experts (i.e. Rubidge for southern Africa [37] and Benton and Surkov for Russia [29]), there are problems with the southern African dataset, because it is largely based on identifications in museum collection records, and does not include some taxa published in the last 10 years. We chose to use each dataset ‘as is’ in order to avoid cherry-picking new taxa from the literature, which biases diversity estimates [51], but we acknowledge this is a limitation of our dataset. Nonetheless, because erroneously identified and new taxa typically represent one or a few specimens, they are unlikely to dramatically affect rarefaction of datasets in the hundreds to thousands of specimens, because they will rarely be picked in the random draws of repeated sub-sampling.

Perhaps the largest obstacle for our dataset is obsolete or changing taxonomy, because certain clades are undergoing major taxonomic revision at the generic level (e.g. procolophonids, therocephalians and *Dicynodon* [47,48,52,53]) that could result in re-identification of significant portions of our datasets. The effects of this taxonomic bias should be partly ameliorated by rarefaction for richness, but it is a significant problem for our unrarefied clade-specific generic richness analysis, so these data should be considered preliminary. This problem should be minimized for evenness, because these revised taxonomies largely affect rarer components of the fauna. In the case of *Dicynodon*, proposed taxonomic revisions [47] will only increase the generic richness and evenness of the Late Permian *Dicynodon* AZ, enhancing observed differences with post-extinction assemblages. The same is true for therocephalian lineages across the extinction interval [53]. In contrast, recent procolophonoid work has increased recognized Triassic diversity, which will reduce the decline we observe in parareptile richness and total raw richness across the boundary. Finally, by definition, genus-level taxonomic problems do not affect our higher clade-level relative abundance results.

Our conservative test of revising the *Lystrosaurus* AZ data bears out these predictions. Major drops in richness and evenness across the Permo-Triassic boundary were still apparent; there was only a slight change in raw richness, and no statistically significant change in rarefied richness, evenness and clade-specific relative abundance (see electronic supplementary material, table S4 and figure S4). The only major change in clade-specific richness is that parareptiles no longer show a major drop across the Permo-Triassic boundary, in agreement with recent discoveries [54]; these changes are expected given our previous prediction that clade-specific richness estimates would be most sensitive to taxonomic and sampling biases. This demonstrates that the major trends in our data are robust to taxonomic error and revision similar to previous results with other datasets [50].

Our analyses are conducted at the genus level because specimen datasets identified to species are not currently available. This means that our analysis could differentially underestimate diversity because some genera are monospecific, whereas others include a great many species. At present, there is no way to correct for this, other than to note that many Permo-Triassic genera are monospecific, and those that contain many species are currently being revised to include one or few species [47]. This limitation affects all richness and evenness values.

To summarize the inherent limitations of our data, we regard our analyses of rarefied generic richness, evenness and clade relative abundance to be the most robust to various biases (figures 2 and 3*b*), though richness is somewhat affected by outcrop area, and both rarefied richness and evenness are moderately affected by taxonomic issues. Raw total and clade-specific generic richness (figures 2 and 3*a*) are sensitive to nearly all of the potential biases described above; thus, we remain fairly conservative in our interpretation of the trends in these data.

Erroneous interpretation can also occur because of poor or incorrect geochronology for temporal bins. This is particularly relevant for our data; although the stages of the Permo-Triassic timescale are well dated [1,40], these radioisotopic ages and the definition of the stages themselves come from the marine record. In contrast, the terrestrial Permo-Triassic is very poorly dated, using non-biostratigraphic means [40], making it difficult to confidently correlate the biostratigraphic zones with the marine timescale. The correlation of the southern African AZs to the timescale [38] is consistent with available radioisotopic ages from the Karoo [55,56], but these U-Pb ages have large analytical uncertainties and are thus not precise enough to pinpoint geochronological boundaries, and some come from the southwestern part of the basin, where there are few vertebrate fossils [56]. As an example of how these correlations might mislead, it is tempting to interpret the low diversity of the *Tropidostoma* AZ in Africa and Malokinelskaya svita in Russia as the immediate after-effects of the end-Guadalupian extinction event (figure 2) [57,58], as did Fröbisch for his dicynodont dataset [36]. Yet the correlation of this AZ to the Permian timescale is only based on long-distance vertebrate biostratigraphic correlations, and could be completely erroneous. It is consistent with Fildani *et al*.'s [56] lithostratigraphic correlation to the better-dated southwestern Karoo Basin, but even these authors noted their lack of confidence in the correlation for this part of the section. Thus, we refrain from making this interpretation at the present time, and note that this uncertainty applies to any previous studies that attempted such a correlation.

There are also arguments over the specific placement of the Permo-Triassic boundary in classic Karoo sections [59], but this should have little effect on our data because the AZs encompass hundreds of metres of section, and few specimens are found within several metres of either side of the generally recognized Permo-Triassic boundary [28,30–32]. Even if some of these specimens are placed on the wrong side of the boundary, they are few, and will be unlikely to affect rarefaction or evenness analyses.

### (b) Palaeoecology of non-marine tetrapods across Permo-Triassic boundary

Our analysis is the first to comprehensively investigate non-marine tetrapod richness, evenness and relative abundance during the end-Permian mass extinction and Triassic recovery using specimen-level data. Despite limitations discussed above, our data clearly demonstrate a major non-marine vertebrate extinction event across the Permo-Triassic boundary, as evidenced by a precipitous drop in rarefied total generic richness and evenness (figure 2). This contrasts with previous conclusions [33–35], but agrees with two recent studies [5,36]. These data also provide clear evidence for a long and delayed recovery. Sample-standardized richness and evenness do not recover, if at all, until the Middle Triassic (figure 2). These results differ strongly from recent conclusions of studies looking at first and last appearances of individual taxa [30–32], and older richness studies [33–35]. Within the available temporal scope of our analysis, it is clear that relative abundance of various clades was permanently changed after the end-Permian extinction (figure 3*b*), in agreement with analyses of ecological guilds [5]. This conclusion is also supported by the permanent drop in dicynodont evenness in southern Africa, and is consistent with the generic richness of individual clades (figure 3*a*), though these data are extremely sensitive to the biases outlined above. In summary, we argue that patterns in richness, evenness and relative abundance from both southern Africa and Russia all point to a major end-Permian extinction event in non-marine tetrapod communities, which did not recover until 5–8 million years later, during the Middle Triassic. Furthermore, the composition of these communities was permanently altered, with the extinction of gorgonopsians, decline in therocephalians, dicynodonts and parareptiles, and rise of temnospondyls, cynodonts and archosauromorphs (figure 3), though future improved sampling and taxonomy may slightly modify these observed patterns.

Prior to full recovery, these post-extinction Early Triassic ecosystems were dominated by a few very abundant forms that are considered ‘disaster taxa’, which took advantage of widespread ecological vacancies. This is exemplified in southern Africa by the extremely common dicynodont *Lystrosaurus*. Our data fully support *Lystrosaurus* as a disaster taxon; its Early Triassic proliferation is evident through the major drop in total and dicynodont evenness across the Permo-Triassic boundary and its widespread geographical range [60,61]; the ubiquity of *Lystrosaurus* is the main reason why dicynodont relative abundance did not change across this boundary (figure 3*b*). The abundant Early Triassic procolophonid parareptile *Procolophon* in southern Africa may have also acted in a similar manner. The low-evenness Early Triassic *Lystrosaurus* AZ, dominated by *Lystrosaurus* and *Procolophon*, is consistent with other terrestrial disaster taxa such as the lycopsid macrofossil *Pleuromeia* (and its possible palynomorph correlates) [19–23,62] and the pteridosperm palynomorphs *Lunatisporites* and *Striatoabieites* [20]. Together, these data suggest low-diversity terrestrial ecosystems dominated by a few taxa that persisted for millions of years after the extinction, similar to evidence from marine ecosystems [8].

Our analysis is particularly novel in investigating evenness, which provides some striking patterns when compared with sample-standardized richness. It appears that in both southern Africa and Russia, evenness recovered to near-pre-extinction levels well before richness did (figure 2). This provides new insight into the ecological recovery of vertebrates on land in the wake of the end-Permian extinction. In particular, we suggest that this pattern is evidence for a two-step recovery, whereby the prevalence of a few disaster taxa is superseded as basic trophic and functional links in the ecosystem are repaired, before the redevelopment of full ecosystem complexity as represented by taxonomic richness. These empirical data match well with simple models of ecosystem recovery from mass extinction [63], which also predict the appearance of disaster taxa and a delayed multi-step recovery, and trophic models suggesting that low evenness makes these Early Triassic ecosystems susceptible to instability [64].

In addition to the occurrence of disaster taxa, richness and evenness patterns from marine invertebrates and non-marine plants also match well with our new data from non-marine vertebrates. Though global analyses of land plant diversity show only a weak to moderate drop in diversity across the Permo-Triassic boundary [62,65], higher-resolution local and regional records of richness and first/last appearances provide strong evidence for a large land plant mass extinction, followed by delayed recovery in the Middle Triassic, millions of years later, and permanent changes in clades that comprise floral assemblages [19,22–24,62,66]. These data compare very well with our non-marine vertebrate record in both magnitude of the extinction, delay in recovery and permanent compositional changes. The same is true for marine records; large-scale global analyses of sample-standardized marine invertebrate palaeodiversity show one of the largest drops in Phanerozoic diversity across the Permo-Triassic boundary, and diversity levels do not even partially recover until the Middle–Late Triassic [3]. Evenness also drops across the boundary, but continues to decline throughout the Triassic [3], in contrast to its recovery in the Middle Triassic for non-marine vertebrates (figure 2). Regional marine invertebrate records show a similar pattern of a major drop in diversity at the Permo-Triassic boundary and a delayed Middle Triassic recovery [6,67], again consistent with our non-marine vertebrate data and published land plant data. Therefore, we can demonstrate unambiguously that the end-Permian mass extinction affected both marine and terrestrial ecosystems in similar ways: a large sudden drop in diversity associated with the extinction itself, low diversity throughout the Early Triassic and recovery during the Middle Triassic.

### (c) Permo-Triassic terrestrial ecosystems and the global carbon cycle

The low Early Triassic richness and delayed Middle Triassic recovery of marine and terrestrial ecosystems (including vertebrates) have been attributed to extrinsic effects such as volcanic activity and/or further greenhouse warming [10,14], because the post-extinction interval is associated with multiple major perturbations of the carbon cycle [6] (figure 2). However, modelling demonstrates that this delayed recovery can be explained largely from intrinsic ecosystem properties [63]. Rather than explaining it as evidence of palaeoenvironmental change, Whiteside & Ward [8] recently proposed that these Early Triassic positive and negative excursion couplets in the carbon cycle (‘chaotic carbon intervals’) record the instability of the ecosystem itself, whereby the abundance of a small number of disaster taxa and low redundancy of trophic links in the ecosystem lead to boom–bust cycles, which are manifested in the carbon cycle through changes in productivity. This hypothesis matches well with the marine invertebrate record [6,8], though there are alternative hypotheses for the correlation between carbon cycling and diversity [7], and differing explanations for this chaotic carbon pattern are not necessarily exclusive. Almost certainly, some component of these fluctuations reflects initial CO_2_ output from flood basalt eruption, and subsequent atmospheric CO_2_ consumption by weathering of these basalts [68]. However, this cannot explain the longer-term multi-million year variation in the carbon cycle, long after basalt eruptions stopped and flows were buried.

Our non-marine tetrapod data are fully consistent with the chaotic carbon hypothesis. The prevalence of disaster taxa, low richness and low evenness in the Early Triassic, along with the low diversity of producers (i.e. plants), would have made this unstable ecosystem susceptible to boom–bust cycles and further extinctions [17,64]. This compares well with available terrestrial carbon isotopic records (figure 2), which, like those from marine strata, display multiple perturbations during the Early Triassic [14,42,43]. These carbon isotope records stabilize during the Middle Triassic, the same time that our non-marine vertebrate data show a full recovery in evenness, partial recovery of richness and major changes in relative abundance (figures 2 and 3). We suspect that the perturbations of the terrestrial carbon cycle reflect at least in part an unstable ecosystem, recorded by the low richness and evenness of Early Triassic non-marine vertebrates and plants. Though we cannot use this correlation to directly infer causation, our hypothesis that post-extinction terrestrial ecosystems suffered from instability as a result of loss of redundant trophic links is strongly supported by ecological modelling of vertebrate food webs for Late Permian and Triassic assemblages from southern Africa [16,17]. These models demonstrate that Early Triassic ecosystems were significantly more unstable than those of pre-extinction assemblages because low diversity and evenness meant fewer redundant trophic links [17], particularly in the disappearance of small to medium-sized herbivores [69]. This fits well with the chaotic carbon hypothesis proposed by Whiteside & Ward [8]. Future comparisons of the non-marine vertebrate record with the carbon cycle to test this hypothesis will benefit from more accurate and precise geochronological resolution, both internal to the study area(s) and in correlation with the marine record.
